# Feedback System Control Optimized Electrospinning for Fabrication of an Excellent Superhydrophobic Surface

**DOI:** 10.3390/nano7100319

**Published:** 2017-10-13

**Authors:** Jian Yang, Chuangui Liu, Boqian Wang, Xianting Ding

**Affiliations:** 1 Institute of Process Equipment, College of Energy Engineering, Zhejiang University, Hangzhou 310027, China; zdhjkz@zju.edu.cn (J.Y.); liucg0982@163.com (C.L.); 2 State Key Laboratory of Oncogenes and Related Genes, Institute for Personalized Medicine, School of Biomedical Engineering, Shanghai Jiao Tong University, Shanghai 200030, China

**Keywords:** feedback system control (FSC), electrospinning, superhydrophobic, optimization

## Abstract

Superhydrophobic surface, as a promising micro/nano material, has tremendous applications in biological and artificial investigations. The electrohydrodynamics (EHD) technique is a versatile and effective method for fabricating micro- to nanoscale fibers and particles from a variety of materials. A combination of critical parameters, such as mass fraction, ratio of N, N-Dimethylformamide (DMF) to Tetrahydrofuran (THF), inner diameter of needle, feed rate, receiving distance, applied voltage as well as temperature, during electrospinning process, to determine the morphology of the electrospun membranes, which in turn determines the superhydrophobic property of the membrane. In this study, we applied a recently developed feedback system control (FSC) scheme for rapid identification of the optimal combination of these controllable parameters to fabricate superhydrophobic surface by one-step electrospinning method without any further modification. Within five rounds of experiments by testing totally forty-six data points, FSC scheme successfully identified an optimal parameter combination that generated electrospun membranes with a static water contact angle of 160 degrees or larger. Scanning electron microscope (SEM) imaging indicates that the FSC optimized surface attains unique morphology. The optimized setup introduced here therefore serves as a one-step, straightforward, and economic approach to fabricate superhydrophobic surface with electrospinning approach.

## 1. Introduction

Superhydrophobic surface, which is defined as a surface with a static water contact angle (CA) greater than 150° and a water sliding angle (SA) smaller than 10° [1], has attracted tremendous interests over the past few years. Some empirical models, such as the well-known Wenzel [2] and Cassie-Baxter [3] models, have been proposed to illustrate surface wetting properties and reveal the relationship between the surface roughness and water repellency. It is well recognized that the combination of suitable surface roughness and low-surface-energy materials is responsible for superhydrophobicity [4]. The unique property is very important for many applications, such as filtration, oil/water separation, self-cleaning, anti-icing, and anti-corrosion [4,5], which in turn drives the relevant research on the fabrication of artificial superhydrophobic surface. Many techniques have been employed to fabricate superhydrophobic surface, such as plasma etching, sol-gel method, template synthesis, phase separation, crystallization control, chemical vapor deposition, and electrohydrodynamics (EHD) [4,5,6].

As one of the most common EHD techniques, electrospinning is considered as the most efficient method to produce fibers with diameters in the range of nanometer, sub-micrometer, and micrometer. The bead-fibers or even particles that formed by electrospinning could potentially build a composite structure with possible superhydrophobic property. Fantini, et al. [7] reported that microspheres or nanospheres could be produced by electrospinning, or called electrospray; as a result of surface tension, the liquid jet prefers to shrink to spherical droplets to acquire the smallest surface area. Jiang et al. [8] has proved the superhydrophobic peculiarities of surfaces made up of a composite structure consisting of porous microspheres and nanofibers by electrospinning, they used polystyrene (PS) solution as precursor solution and successfully fabricated hierarchical microbead-fiber structure via electrospinning technology by changing the solution concentration individually. For its high roughness and low surface energy properties of the PS material itself, the fabricated membrane shows an excellent superhydrophobic property. The process of electrospinning is also easy to set up for laboratory research, as it offers many controllable parameters that can be used to obtain the required surface morphology, such as applied voltage, distance of collector, concentration of the solution, the choice of solvent, and pinhole size, etc., all of which have their respective influence on the fabricated film [9]. As the surface morphology is the main factor for determining superhydrophobic property, just like the micro topology of lotus leaves, which have double scale roughness microstructure and low surface energy epicuticular wax [10]. Therefore, superhydrophobic properties of the electrospun membrane can be generated by adjusting different processes and material parameters.

From the current research point of view, the researchers mostly studied the influence of single parameter by fixing other experimental parameters, and ignored the integrate influences to the structure of the fabricated electrospun membranes between the different parameters. In this study, we will change more than one parameter simultaneously to study the integrate influence of different parameters by algorithm, and meanwhile to search for an optimal parameter combination quickly to fabricate an excellent superhydrophobic surface by one-step electrospinning without any further modification. However, instead of testing all of the possible combinations of these parameters, we applied the feedback system control (FSC) platform to systematically search for optimal parameter combinations. The platform was based on the integration of experimental results and a feedback search guided by the differential evolution (DE) algorithm and stepwise regression. With the FSC platform, only a few possible combinations are needed to derive effective combination from a vast search space. The FSC technique [11,12,13,14] focuses on a desired phenotypic output, such as the superhydrophobic property of the fabricated membrane by electrospinning, as in this study, rather than on detailed mechanism hypothesis on factor-factor interactions.

## 2. Materials and Methods

### 2.1. The FSC Platform

**Figure 1:**
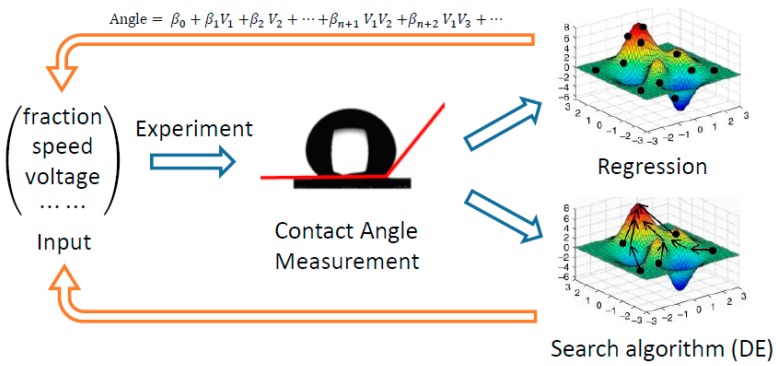
Schematic diagram of Feedback System Control (FSC) framework for optimization of the contact angle.

The feedback system control scheme (FSC), based on the closed-loop feedback control process, mainly consists of five components, as outlined in Figure 1, including input, experiment, contact angle measurement, search algorithm, and regression. The input is the value of each parameter in each combination. The initial parameter combinations were generated according to a Latin Square design of experiment, while the other combinations were chosen by the search algorithm and regression model. Then the membranes were fabricated under the condition of the defined parameter combinations by one-step electrospinning, and subsequently the static water contact angle of the electrospun membranes was measured. Contact angle is selected to provide a phenotypic output that is used to assess the influence of the parameter values and its combinations on the superhydrophobic property. The FSC optimization is thus based on an integrative system, wherein the difference between the desired and real system response is used as optimization criterion to be fed into a search algorithm (DE) [15,16]. The search algorithm therefore uses the difference between the results of the parameter combinations and the desired optimization goals to predict new combinations. Meanwhile, a regression method for input-output analysis was used to aid in the prediction of new combinations. Interactively, the FSC framework is characterized by iterative experiments and predictions from both regression and search algorithm.

### 2.2. Design of Experiment

**Figure A1:**
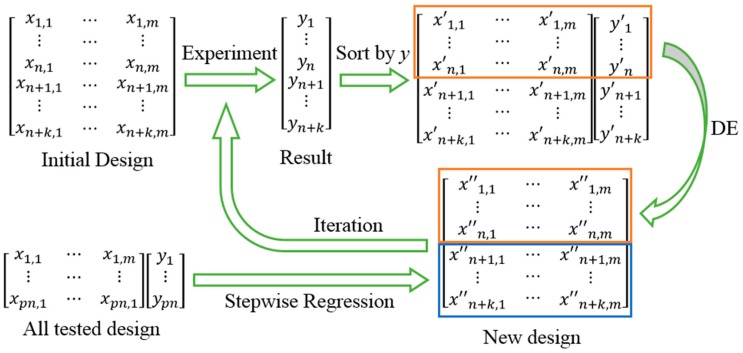
A mathematical diagram of the FSC framework featuring the integration of DE and stepwise regression. The experiments are conducted iteratively. The initial combinations are designed by Latin Square. Each column of the matrix represents a parameter variable and each row represents an experiment of combinatorial parameters. $x_{i,j}\left(i=1,\;2,\;\ldots ,n+k;j=1,\;2,\ldots ,m\right)$ represents the level of each parameter. The optimization target, in this case contact angles, are measured via experiments and denoted as $y_{i}\left(i=1,2,\ldots ,n+k\right)$. After each iteration of experiments, the order of the rows in the matrix containing all tested combinations are sorted by $y_{i}$ descendingly. The first *n* + 2 combinations are used to generate *n* new combinations by the DE algorithm where in $x_{i,j}^{'}=x_{i,j}+M\left(x_{i+1,j}-x_{i+2,j}\right)$. The rest *k* combinations are generated according to prediction of stepwise regressing all tested combinations. In this study, *n*, *k* and *m* are 7, 3 and 7 respectively, while they can be flexibly adjusted according to different optimization application.

The optimization of contact angle was performed iteratively, wherein the design of every iteration except for the initial one was determined by the contact angle measurement result of previous iterations (Figure A1). Each iteration contains ten combinations of the seven condition variables, including mass fraction, mass ratio of N-Dimethylformamide (DMF) and Tetrahydrofuran (THF), inner diameter of needle, feed rate, receiving distance, applied voltage, and temperature with 5, 5, 5, 4, 4, 4, and 4 levels, respectively. Except for the initial iteration, seven of the ten combinations in each iteration were obtained from the DE algorithm and three were from regression modeling. PS (Polystyrene, *Mw* = 260,000, J & K Scientific LTD., Beijing, China) was separately dissolved in DMF (Sinopharm Chemical Reagent Co., Ltd., Beijing, China) or THF (Sinopharm Chemical Reagent Co., Ltd., Beijing, China) or both with a certain mass ratio by stirring for 4 h to form five levels of mass fraction transparent solutions. The precursor solution was placed in a 20-mL syringe equipped with a blunt metal needle, and the inner diameter of the needle contains five different values. The syringe was placed in a syringe pump at 4 feed rates respectively. A stainless-steel cylinder covered with a sheet of aluminum foil was employed as the collector. The cylinder had a fixed rotation speed about 160 r/min. The distance between the needle tip and the collector was set at five different values. The applied voltage and ambient temperature were also set at four different values. Other parameter settings remained consistent, including the solute mass of fabricating each sample and the humidity in the laboratory, which was fixed at 30%. The specific values of these parameters that are considered in this study are shown as Table 1.

### 2.3. Search Algorithm (DE)

**Figure A2:**
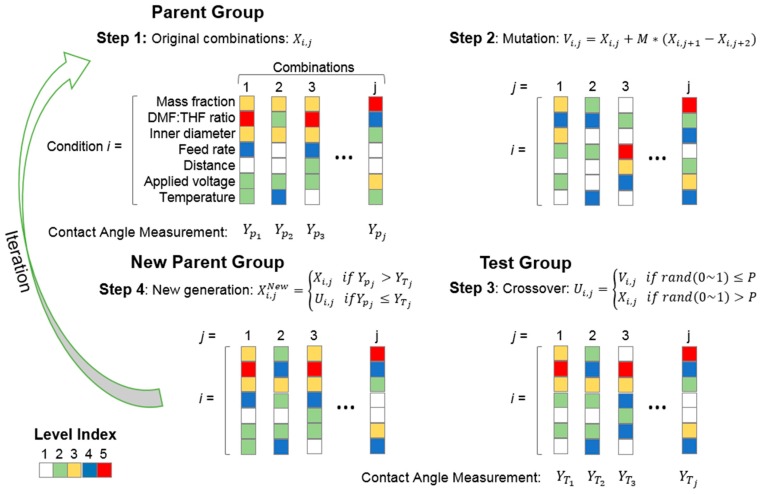
A schematic diagram of DE algorithm. The algorithm goes through four steps in each iteration of the experiments. (**1**) Generation of the parent group. In the first iteration, the parent group is not generated by the DE algorithm. Originally, a set of random combinations is often adopted, while in this study, we use the Latin Square design of experiments to improve the optimization efficiency. As shown in this diagram, the color of each square represents the level of the parameter, and each column represents a parameter combination. (**2**) Mutation is introduced to each square based on subsequent combinations with the Mutation Constant M. (**3**) Generation of test group by crossover with Crossover Constant P. For each square in the new test group, a random number is used to determine whether the mutation is reserved. After the crossover, the new test group is tested via experiment. (**4**) Generation of new parent group. The experimental result of each combination in the test group is compared with its according combination in the parent group, and the one with better performance is reserved for the new parent group. The output of step 4 will be the input of step 1 after the second iteration, and these steps loop until the optimal combination is found.

The DE algorithm was coded with MATLAB software according to Storn and Price’s [16] original design (Figure A2). Each combination of condition variables was represented as a vector in the algorithm. The value of each variable was transformed into integer codes rather than its real value in this software. All ten combinations of the initial iteration were designed based on a Latin Square [17] for evenly distributing limited number of combinations in the combinatorial spaces.

### 2.4. Regression Model

**Figure A3:**
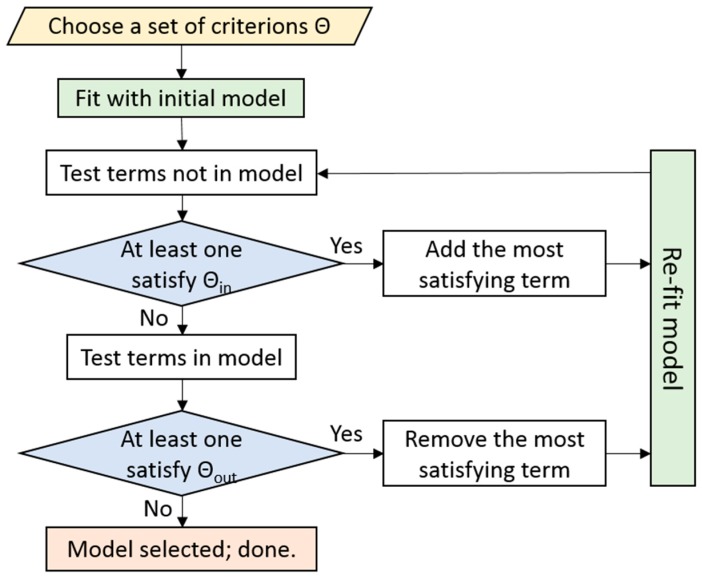
A schematic diagram of stepwise regression. Stepwise regression fits polynomial models iteratively. First, a set of criterions is chosen to determine whether to add terms or remove terms. Then, the fitting starts with an initial model, usually a constant model or a linear model. After that, terms that are not in the model are tested separately. If there is at least one term that satisfies the addition criterion, the most satisfying term will be added into the model, and the renewed model will be fit again. The addition step loops until there is no term not in the model that satisfies the addition criterion. After addition of terms, all the terms in the model are tested with the removal criterion. The removal step is similar to the addition step, but differs in that once a term is removed from the model, the model is fit again, and the regression process is returned to the addition step. The whole regression process ends when there are neither terms that can be added into the model nor terms that can be removed from the model.

Stepwise regression was adopted to model the relationship of each condition variable as well as their interactions with the contact angle. The algorithm was fulfilled by the built-in function ‘stepwiselm’ in MATLAB 2016b software, with the logic demonstrated in Figure A3. All of the input variables were normalized to interval (0, 1) by being divided by their maximal level. The Akaike information criterion (AIC) was used to determine whether to add or remove terms. The regression started with only a constant term and was limited to add only linear terms and interaction terms.

### 2.5. Characterization

The morphology of membrane observed by Thermal Field Emission Scanning Electron Microscope (FSEM, SIRION-100, FEI, Hillsboro, OR, USA) required an ion coating with gold by a sputter coater for 300 s under vacuum at a current intensity of 15 mA after mounting the sample on metallic studs with double-sided conductive tape. The accelerating voltage during scanning was 25 kV. Water contact angle (CA) was measured by Optical Contact Angle Measuring Device (OCA20, DATAPHYSI, Stuttgart, Germany) at ambient temperature. We measured each sample five times and removed a maximum and a minimum from the five values, and then calculated the average value of the remaining three values. When considering that the contact angle measurement is influenced by the volume of the water droplet and the gravity force [4], we used a consistent volume of water droplet about 3.5 μL to measure CA. Sliding angle (SA) was determined by placing a water droplet (10 μL) on the surface of membrane, which was then gradually inclined until the water droplet began to roll.

## 3. Results and Discussion

Making a rough substrate and meanwhile modifying it with low energy materials is the main method to make superhydrophobic surface. EHD technique has proved to be an effective yet simple method for fabricating microsphere/nanofiber composite film with a very rough surface by one-step without any modification. Here, by adjusting the seven parameters that mainly impact the morphology of membrane, we search for an optimal condition quickly via the FSC platform that could fabricate electrospun membrane with enough roughness to achieve a best performance about superhydrophobic property. In this study, since the contact angle of the electrospun membrane was the sole objective function used in the FSC screening for different parameter combinations, 10 initial parallel combinations with arbitrarily chosen values were generated using the numerical analysis software MATLAB. As the FSC progressed, the 10 parameter combinations were updated based on the measured CA values of the electrospun membranes.

The first round of combinatorial experiments is marked as Iteration 0, since the initial combinations were not generated by our optimization approaches. The optimization process started at the second round, marked as Iteration 1 and stopped at the fourth round marked as Iteration 3 where several good-performance combinations have already been found. In addition, we also performed the fifth round of experiments including only six combinations for testing our optimization and modeling methods, which was marked as Final Iteration (Iter.F). In each iteration, seven of the ten combinations were generated by the DE algorithm with data of nine best rank combinations in the previous round of experiments and the other three combinations were generated from the regression model using all the available combinatorial data. The Mutation Rate (M) and Crossover Rate (P) in the DE algorithm were separately 0.75 and 0.75 for higher evolution rate so that optimal combinations could be found in limited iterations (Figure A2). Stepwise regression was chosen as the modeling approach for its flexibility and self-adaptability on dealing with both small and large data volumes (Figure A3). To avoid overfitting, we adjusted the thresholds of the term adding and removing criterion in stepwise regression where the number of terms in the model was less than the number of data points. The model for predicting each iteration of experiments are all well regressed with the correlation coefficient, R, greater than 0.9 and well distributed residues (Figure 1c).

**Figure 2:**
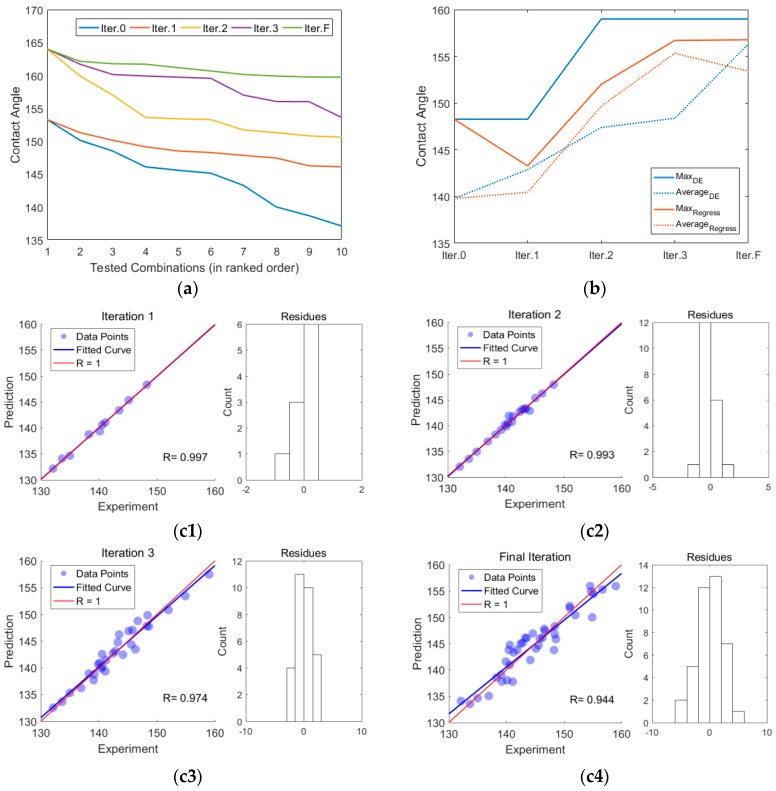
Optimization of contact angle in five rounds of experiments. (**a**) Best 10 combinations found in five rounds of experiments are represented by five colored lines. (**b**) Contact angles of the best combination and average performance by regression and differential evolution (DE) separately in each round of experiments. (**c**) Correlation coefficient and distribution of SSE (sum of squares for error) for the regression model used to predict each iteration.

As shown in Figure 2a, with the integration of DE and stepwise regression, the contact angle was optimized iteratively. The optimal combination was found in the third round of experiments, but in the subsequent iterations more combinations with acceptable contact angles were discovered for further characterization afterwards. From the first round of iteration represented by blue line, to the final round of iteration represented by green line, the contact angle as the objective function value had a growing trend; furthermore, the line of each round became smoother as the FSC progressed and reached a relative plateau at the final round, of which the CA values are all larger than 155°.

Figure 2b demonstrated the contribution of the DE algorithm and regression in the whole optimization process. At first, with limited number of data points (Iter.1), the regression model had a poor performance resulting in the fact that the best prediction was inferior to the best of the initial design (Iter.0). However, although the optimum was identified by DE in Iter.2 by chance, the performance of regression modeling improved steadily in Iter.2 & 3 with more data points involved. Predictions of regression modeling provided a guideline for the stochastic search of the DE algorithm, therefore making the average contact angle of DE-generated combinations greater than regression modeling again in the end (Iter.F).

**Figure 3:**
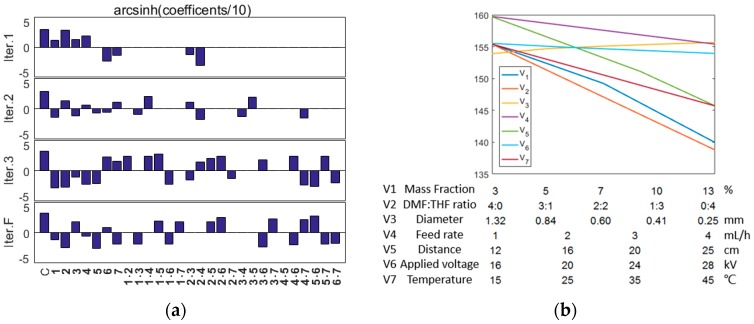
Coefficients of every term in the regression models corresponding to Figure 1c. (**a**) From left to right, C and bold numbers are separately representing the constant term, linear terms (1~7: mass fraction, mass ratio of N-Dimethylformamide (DMF) and Tetrahydrofuran (THF), inner diameter of needle, feed rate, receiving distance, applied voltage and temperature), and interaction terms. The y coordinates are transformed by a non-linear function arcsinh (x/10). (**b**) Each line represents the variation of contact angle caused by change of each single condition predicated by model of Iter.F in the optimal combination identified, and the labels V1 to V7 at the bottom give the values of the different x-axes for the data plotted on the y-axis as V1 to V7.

This integrative platform of DE and stepwise regression seems to be more efficient than conventional FSC platforms 14 in identification of optimal combinations. Moreover, the regression model can tell us the contribution of each parameter and their interactions to the CA value (Figure 2) by evaluating the coefficients. With more data points acquired, the regression model has gone through an evolution process (Figure 2a), wherein the contributions of some terms disappeared and some emerged in each iteration, making the model much more robust. To directly demonstrate the contribution of the parameters, we adopted the model of the Final iteration to predict how each single parameter and their interactions would affect the CA value at the optimum state. It can be seen from the bottom term of Figure 3a that the interactions between these seven parameters have different intensity effect on the CA values. Figure 3b shows that lower mass fraction, THF ratio, feed rate, receiving distance, and temperature may benefit the CA value while the nozzle diameter and applied voltage have little effect.

**Figure 4:**
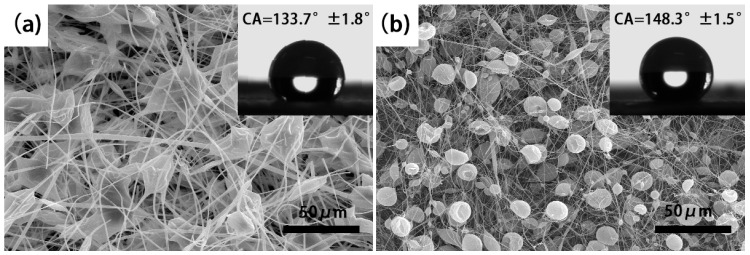
SEM images of the worst (**a**) and best (**b**) hydrophobic membranes in the initial round.

For the purpose of verifying the optimization process from the perspective of microstructure, the change process of the morphologies of electrospun membranes can be observed. In the initial round, the CA values of all fabricated electrospun membranes are smaller than 150°, while some are even smaller than 140°. SEM images indicated that all of them are hierarchical microbead-fiber structure, which can maintain air entrapped in the bulk layers. By providing continuous water-air-solid interfaces, it can keep a stable state of superhydrophobicity. The differences between them are the shape and size of beads and the diameter of fibers. Figure 4 shows the morphologies of the best and worst hydrophobic membranes in the initial round. Obviously, the bead and the diameter of fibers of Figure 3a are larger than Figure 4b, and the bead of Figure 4a shows an irregular shape while of Figure 3b has a very uneven size distribution that range from 2 μm to 12 μm. However, the smaller structure has a better hydrophobic performance.

**Figure 5:**
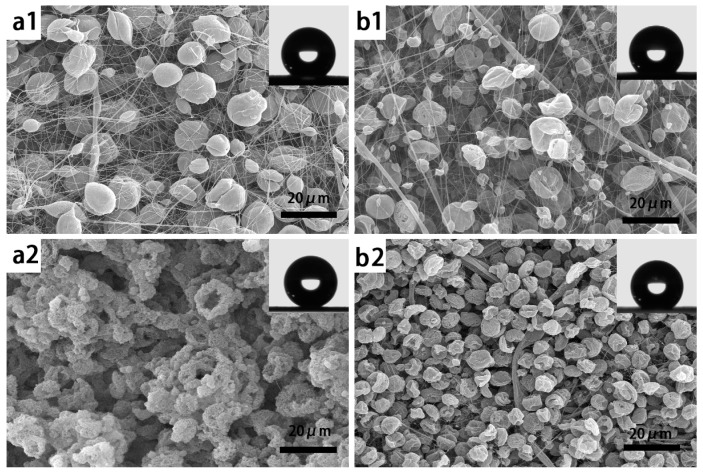

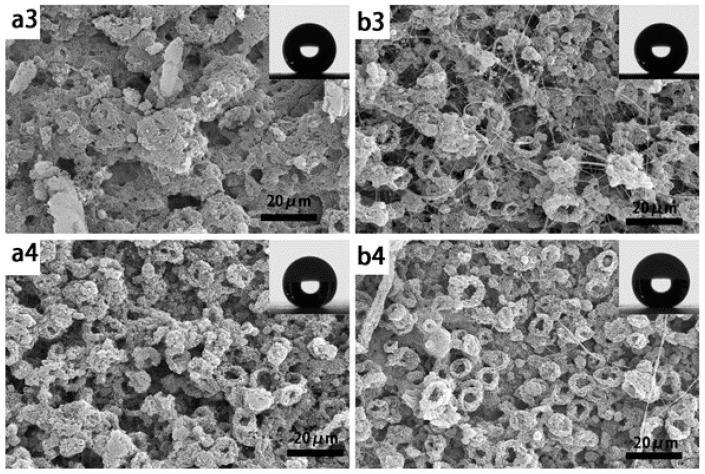
SEM images of the best combination by DE and regression in each round of experiments. (**a1**–**a4**) From a1 to a4, the SEM images show the morphology of electrospun membranes, which have the largest CA value of Iter.1, Iter.2, Iter.3 and Iter.F predicted by the DE algorithm separately. (**b1**–**b4**) From b1 to b4, the SEM images show the morphology of electrospun membranes, which have the largest CA value of Iter.1, Iter.2, Iter.3 and Iter.F predicted by regression separately.

**Figure 6:**
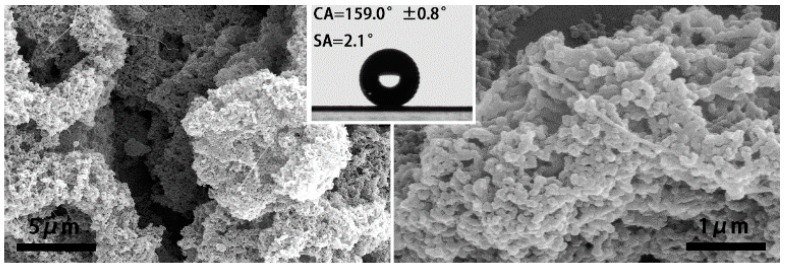
SEM image of the membrane fabricated under the condition of best parameter combination in all experiments.

Then, after operating several rounds of experiments that were predicted by DE and regression, the structure of the membranes became much smaller and rougher to get a much better performance on hydrophobicity. As shown in Figure 5, (a1) and (b1) show the morphologies of electrospun membranes, which have the best hydrophobic performance (CA = 146.3° and 143.3°) of the first round predicted by DE and regression separately. It can be seen that they are both composite membrane with a hierarchical microbead-fiber structure. (a2) and (b2) are the best (CA = 159.0° and 152.0°) in the second prediction round. When compared to (a1) and (b1), (a2) and (b2) both scarcely have fibers. As the same as (a3), (b3) (CA = 155.1° and 156.7°) and (a4), (b4) (CA = 156.8° and 157.1°), the particles or irregular shape clusters are the main structure of the electrospun membranes. Especially, the irregular shape cluster has a very rough surface that has a much smaller magnitude. It can be observed that from Figure 6, a rough surface with a nanoscale structure was formed, combined with the microscale gaps between clusters, resulting in creating enhanced surface roughness and additional surface area, which significantly improves the superhydrophobic property. With the CA value much larger than 150°, the water sliding angle (SA) is very small, about 2.1°, that is much smaller than 10°.

## 4. Conclusions

In this study, an optimal parameter combination was quickly and successfully identified via the FSC platform with the integration of DE and stepwise regression to fabricate an excellent superhydrophobic surface with CA values reaching up to 159.0° and SA smaller than 3° by one-step electrospinning without any further modification. Five rounds of experiments were conducted. The first four rounds of experiments included 10 combinations, while the final round of experiments included only six combinations, which was used to test our optimization and modeling methods. In each iteration except the first one, seven of the ten combinations were generated by the DE algorithm with data of the best rank combinations in the previous round of experiments, and the other three combinations were generated from the regression model using all of the available combinatorial data. After four rounds of iterations, it can be found that several good-performance combinations with CA values of the fabricated electrospun membranes are larger than 155°. With the final iteration, the regression model also determined the contribution of each parameter and their interactions to the CA value that lower mass fraction, THF ratio, feed rate, receiving distance, and temperature may benefit the CA value while the pinhole size and applied voltage have little effect. In conclusion, this work provides an efficient method to investigate the integrated influence of different parameters on the property of electrospun membrane and quickly search for an optimal parameter combination to fabricate excellent superhydrophobic surface by electrospinning, by which only a few rounds of experiments are needed from a vast search space with multiple search parameters.

## Figures and Tables

**Table 1:** The specific values of different parameters.

No.	Parameters	Values				
V1	Mass fraction	3%	5%	7%	10%	13%
V2	DMF:THF	4:0	3:1	2:2	1:3	0:4
V3	Inner diameter (mm)	1.32	0.84	0.6	0.41	0.25
V4	Feed rate (mL/h)	1	2	3	4	
V5	Distance (cm)	12	16	20	25	
V6	Applied voltage (kV)	16	20	24	28	
V7	Temperature (°C)	15	25	35	45	

## Supplementary Materials

The following are available online at http://www.mdpi.com/2079-4991/7/10/319/s1, MATLAB code S1: Iteration.
