# Supplementary material for: Feedback System Control Optimized Electrospinning for Fabrication of an Excellent Superhydrophobic Surface
Source: Nanomaterials (Basel). 2017 Oct 13;7(10):319. doi: 10.3390/nano7100319 (PMC5666484; doi:10.3390/nano7100319)
Supplement: Supplementary file 1 [file nanomaterials-07-00319-s001.pdf]

Feedback System Control Optimized Electrospinning for Fabrication of an Excellent Superhydrophobic Surface

Jian Yang [1], Chuangui Liu [1], Boqian Wang [2,*], Xianting Ding [2,*], Mian Li [3] and Deshui Cheng [3]

MATLAB code S1: Iteration

```
clear

load data


N=1; % Iteration

iter_data=iter_data(1:N);

M=0.75; P=0.75; %parameters for DE


% DE for first 7 trials

if N~=1

   temp=cell2mat(iter_data');

else

   temp=iter_data{1,N};

end


% pre-process

temp=flip(sortrows(temp,8)); % sort by result

X_dig=temp(:,9:15);

clear temp


temp=X_dig(1:7,:)+M*(X_dig(2:8,:)-X_dig(3:9,:)); % Mutation

temp=round(temp);

temp(temp(:,1)<1,1)=0;% add 0 in column 1
```

```
temp(temp(:,1)>4,1)=4;% remove 5 in column 1

temp1=temp(:,2:7);

temp1(temp1<1)=1; % >1 in column 2:7

temp(:,2:7)=temp1;

% temp(temp<1)=1;

temp1=temp(:,1:3); % <5 in column 1:3

temp2=temp(:,4:7); % <4 in column 4:7

temp1(temp1>5)=5;

temp2(temp2>4)=4;

temp=[temp1 temp2];

temp(temp(:,2)>4,2)=4; % no 0:4

clear temp1 temp2


pp=rand(7,7); %crossover

X_dig=X_dig(1:7,:);

temp(pp>P)=X_dig(pp>P);

temp(temp(:,2)>4,2)=4; % no 0:4


temp(:,1)=temp(:,1)+ones(size(temp(:,1)));% add 3 to v1

x1=cell(7,7);

for i=1:7

   x1(:,i)=map(temp(:,i),i);

end

temp(:,1)=temp(:,1)-ones(size(temp(:,1)));% add 3 to v1


% Regression for last 3 trials

x=[];

for i=1:N
```

```
    x=[x; iter_data{1,i}];
end


X=x(:,1:7)./repmat(MAX,[10*N 1]); % X data normalization
Y=x(:,8);
%%
mdl=stepwiselm(X,Y,'interactions')%,'criterion','aic','penter',0.05,'prem
ove',0.115)
%%
%result=zeros(5*5*5*4*4*4*4,8);
result=[];


for i=1:7
    %value{i}=unique(X(:,i))';
    eval(['v' num2str(i) '=unique(X(:,' num2str(i) '));']);
end
v1=[0.2;0.333333333333333;0.466666666666667;0.666666666666667;0.866666666
666667];
parfor i1=1:5
    i1
    for i2=1:4
        for i3=1:5
            for i4=1:4
                for i5=1:4
                    for i6=1:4
                        for i7=1:4
                            input=[v1(i1) v2(i2) v3(i3) v4(i4) v5(i5) v6(i6)
v7(i7)];
                            result=[result; [input predict(mdl,input)]];
```

```
                        end
                    end
                end
            end
        end
    end
end
clear v1 v2 v3 v4 v5 v6 v7
result=flip(sortrows(result,8));
```
